# Public Response to Obamacare on Twitter

**DOI:** 10.2196/jmir.6946

**Published:** 2017-05-26

**Authors:** Matthew A Davis, Kai Zheng, Yang Liu, Helen Levy

**Affiliations:** ^1^ University of Michigan School of Nursing Ann Arbor, MI United States; ^2^ Donald Bren School of Information and Computer Sciences University of California Irvine, CA United States; ^3^ University of Michigan School of Information Ann Arbor, MI United States; ^4^ University of Michigan Institute for Social Research Ann Arbor, MI United States

**Keywords:** Patient Protection and Affordable Care Act, health care reform, social media, data collection

## Abstract

**Background:**

The Affordable Care Act (ACA), often called “Obamacare,” is a controversial law that has been implemented gradually since its enactment in 2010. Polls have consistently shown that public opinion of the ACA is quite negative.

**Objective:**

The aim of our study was to examine the extent to which Twitter data can be used to measure public opinion of the ACA over time.

**Methods:**

We prospectively collected a 10% random sample of daily tweets (approximately 52 million since July 2011) using Twitter’s streaming application programming interface (API) from July 10, 2011 to July 31, 2015. Using a list of key terms and ACA-specific hashtags, we identified tweets about the ACA and examined the overall volume of tweets about the ACA in relation to key ACA events. We applied standard text sentiment analysis to assign each ACA tweet a measure of positivity or negativity and compared overall sentiment from Twitter with results from the Kaiser Family Foundation health tracking poll.

**Results:**

Public opinion on Twitter (measured via sentiment analysis) was slightly more favorable than public opinion measured by the Kaiser poll (approximately 50% vs 40%, respectively) but trends over time in both favorable and unfavorable views were similar in both sources. The Twitter-based measures of opinion as well as the Kaiser poll changed very little over time: correlation coefficients for favorable and unfavorable public opinion were .43 and .37, respectively. However, we found substantial spikes in the volume of ACA-related tweets in response to key events in the law’s implementation, such as the first open enrollment period in October 2013 and the Supreme Court decision in June 2012.

**Conclusions:**

Twitter may be useful for tracking public opinion of health care reform as it appears to be comparable with conventional polling results. Moreover, in contrast with conventional polling, the overall amount of tweets also provides a potential indication of public interest of a particular issue at any point in time.

## Introduction

Americans have strong opinions about health care reform. Polls of the general public consistently indicate that less than half of Americans support the Affordable Care Act (ACA) [1]. The ACA (or “Obamacare,” as it is more often called) is a federal statute that was enacted under President Barack Obama in 2010. Among the most significant health care reform efforts in US history, the ACA contains a series of provisions that have been implemented since it was signed into law. The overarching goal of the ACA is to expand access to affordable insurance coverage. However, the ACA remains controversial among policymakers and the general public. Although individual elements of the law, such as subsidies for low-income families to purchase health insurance or the individual mandate, may be more or less popular than the law as a whole [2], the public opinion regarding the law has been surprisingly stable in the 5-plus years since the it was enacted. This stability has endured despite numerous ups and downs in the ACA’s fortunes, including three major Supreme Court decisions (one mostly affirming and the other entirely affirming the law, with a third dealing its supporters a blow) and a botched rollout of the law’s signature initiative, private health insurance exchanges. Table 1 presents a timeline of key events in the implementation of the ACA’s coverage provisions [3-7].

**Table 1:** Timeline of key events related to the implementation of the Affordable Care Act.

Date	Event
March 23, 2010	ACA^a^ signed into law by President Obama. Key coverage provisions—Medicaid expansion and health insurance exchanges—are scheduled to take effect in January 2014. Multiple lawsuits challenging different provisions of the law are filed shortly after its enactment.
2010-2011	Early ACA provisions are implemented, including consumer protections (eg, prohibitions on annual and lifetime caps on coverage) and the requirement that employer-sponsored plans must offer coverage for dependent children up to age 26 years. Most of these take effect as private plans were renewed; as a result, they do not have a single “headline” date for implementation.
December 19, 2011	The SCOTUS^b^ announces it will hear oral arguments in NFIB^c^ versus Sebelius, challenging the constitutionality of two key ACA provisions: the requirement that all individuals have coverage (the “individual mandate”) and the expansion of Medicaid to all individuals with incomes below 138% of poverty.
March 26-28, 2012	The SCOTUS hears oral arguments in NFIB versus Sebelius, generating tremendous speculation.
June 28, 2012	The SCOTUS rules in NFIB versus Sebelius. The individual mandate is affirmed whereas the Medicaid expansion is effectively rendered optional for states: a mixed decision, but on balance regarded as a win for the ACA.
November 6, 2012	President Barack Obama reelected.
October 1, 2013	The first open enrollment period begins for private health insurance exchanges; the federal exchange website healthcare.gov fails to work properly, generating negative publicity.
November 26, 2013	The SCOTUS announces it will hear oral arguments in Burwell versus Hobby Lobby, challenging a private employer’s refusal on religious grounds to provide full insurance coverage for contraception.
January 1, 2014	Expanded coverage through health insurance exchanges starts.
March 31, 2014	The first open enrollment period ends.
March 25, 2014	The SCOTUS announces it will hear oral arguments in Burwell versus Hobby Lobby, which is about whether corporations owned by religious families can refuse to comply with an ACA requirement that their health insurance must fully cover contraception for female workers.
June 30, 2014	The SCOTUS rules in favor of the corporations in Burwell versus Hobby Lobby (a blow to the ACA).
November 8, 2014	The SCOTUS announces it will hear oral arguments in King versus Burwell, which challenges the payment of federal subsidies for health insurance in states that rely on healthcare.gov (a majority of states).
November 15, 2014	The second open enrollment period begins for private health insurance exchanges; healthcare.gov works as intended.
February 15, 2015	The second open enrollment period ends for private health insurance exchanges.
March 4, 2015	The SCOTUS hears oral arguments in King versus Burwell.
June 25, 2015	The SCOTUS Court rules in favor of the Obama administration in King versus Burwell.

^a^ACA: Affordable Care Act.

^b^SCOTUS: Supreme Court of the United States.

^c^NFIM: National Federation of Independent Business.

Public opinion may have briefly dipped or risen immediately after these key events [8,9], but at the time of this writing, the law’s favorable and unfavorable ratings in the Kaiser health tracking poll both stand at 42% —statistically indistinguishable, given the poll’s ±3% point margin of error, from the 46% favorable or 40% unfavorable ratings the law had in April 2010, weeks after it was first enacted [10].

Monitoring public response to new laws and regulations, such as those included in the ACA, is of considerable interest to health policymakers, government agencies, and the media. Traditionally, measuring public response has relied on expensive and time-consuming surveys administered by polling agencies including the Pew Research Center and the Kaiser Family Foundation. Changes in technology introduce new opportunities for tracking public response. One particularly rich source of data is Twitter. Twitter has been used to study natural disasters [11,12], infectious disease outbreaks [13,14], drug and alcohol use [15,16], and public responses to health policies [17-19]. Whereas use of social media data has some limitations [20], it is inexpensive, immediate, and can offer contextual insights not captured by traditional survey questionnaires.

The aim of this paper was to examine the extent to which Twitter data can be used to measure public response to the rollout of the ACA. Our specific research questions were: (1) To what extent can ACA-related tweets be accurately identified? (2) Does the overall volume of ACA-related tweets respond to key events in the implementation of ACA? (3) Is there an association between public opinion (ie, favorable vs unfavorable) measured using ACA-related tweets and conventional polling data from the Kaiser Family Foundation health tracking poll? and (4) What are common words used in favorable versus unfavorable ACA-related tweets?

## Methods

### Twitter Data

We examined the extent to which Twitter data can be used to measure public response to the ACA over time. To do so, we identified relevant tweets over a 6-year time period, examined them, and compared the ACA-related tweet sentiment with conventional polling data of public opinion. This study used publicly available data for all analyses and was deemed to be exempt from institutional board review.

We prospectively collected a 10% random sample of daily tweets (approximately 52 million since July 2011) using Twitter’s streaming API (ie, the “Twitter Gardenhose”) from July 10, 2011 to July 31, 2015. All analyses were restricted to English-language tweets.

To identify tweets about the ACA in this sample, we developed a list of key search terms. From the ACA Wikipedia page [21] and an arbitrary sample of comments to ACA lay media articles, we examined word frequencies in order to identify common text expressions related the ACA. Next, we used Google Trends to identify other words associated with Google searches about the ACA [22]. Based on the collection of terminology used across these data sources, we developed a list of common words and phrases to be used in our search (Textbox 1). Using regular expressions for our search terms (which account for differences in capitalization and spelling), we identified a tweet as being ACA-related if it included any of these words or phrases [23]. This resulted in a sample of 3,300,648 ACA-related tweets.

We also used Twitter hashtags to expand our identification of tweets about the ACA (Textbox 2). A Twitter hashtag is a short phrase that assigns a tweet to a specific topic. The inclusion of tweets identified exclusively based on ACA hashtags resulted in an addition of 75,133 tweets. Thus our final sample comprised 3,375,781 tweets potentially related to the ACA.

To check the validity of this method for identifying tweets related to the ACA we pulled a random sample of 1000 tweets. Two separate members of the research teach reviewed each tweet to determine if it was indeed relevant to the ACA. Thirty-seven of these tweets were not in fact ACA-related (in several the tweet in question used the term ACA as an abbreviation for “acapella” and the tweet was related to singing; Table 2). Therefore, we conclude that our identification strategy had a positive predictive value of 96.3%.

**Search terms used to identify tweets about the Affordable Care Act.**

Terms used in tweets

• Affordable Care Cct or ACA

• Healthcare insurance exchanges

• Healthcare reform act or bill

• Healthcare insurance act or bill

• Obamacare

• Patient Protection and Affordable Care Act or PPACA

**Hashtags used to identify tweets about the Affordable Care Act.**

Hashtags

• #ACA

• #aca

• #Obamacare

• #ObamaCare

• #obamacare

**Table 2:** Selected examples of relevant and nonrelevant Affordable Care Act-related tweets.

Type	Examples
Relevant favorable ACA^a^ tweets	“Finally, my two favorite things come together: online shopping and buying health insurance.”
	“In response to Obamacare, nearly 1 in 3 health facilities are adding doctors.”
	“The GOP Is Terrified Obamacare Could Be a Success.”
	“Thanks to the ACA, Over 5800 Californians with Pre-Existing Conditions Now Getting Care.”
	“Obamacare winning one step at a time, sometimes take double steps. Today, good news for people with heart disease.”
Relevant unfavorable ACA tweets	“Dems Throwing Granny Off the Cliff: Obamacare Cuts Medicare, Seniors Losing Doctors.”
	“4 Years Later ObamaCare Still a Crime Against Democracy That The American People Will Never Accept.”
	“Obamacare: Biggest Job-Killing TAX in US History!”
	“The people of America have no concept at this point as to just how miserable Obamacare is going to make individual lives.”
	“Weird new error screen for Obamacare.”
Nonrelevant ACA tweets	“You’re one of those acapella girls, I’m one of those acapella boys, and we’re gonna have aca-children.”
	“Who’s watching the ACA’s?!”
	“Thank you guys so much for last night. The aca awards were a blast. Thanks for making 2013 unbelievable.”

^a^ACA: Affordable Care Act or acapella.

### Sentiment of Affordable Care Act (ACA) Tweets

We used standard text sentiment analysis to assign each ACA tweet a measure of positive to negative sentiment. Text sentiment analysis uses a lexicon of words each with previously assigned numeric measures of emotion (ranging from negative to positive, ie, −1.0 to +1.0). In this study, tweet sentiment was measured using labMT, a lexicon developed by Dodds et al based on human review of terms from language used in Twitter, Google Books, music lyrics, and the New York Times) [24]. This lexicon has been widely used in studies of Web-based product reviews and temporal patterns of happiness.

After tweets were processed to remove words that do not convey specific content (such as “a” or “the”), the assigned scores for words in a given text were summed up to arrive at an overall score of the sentiment. Tweets with a sentiment score greater than zero were coded favorable while those with a score less than zero were coded unfavorable.

### Kaiser Family Foundation Health Tracking Poll

Since the enactment of the ACA in March 2010, the Kaiser Family Foundation’s health tracking poll has been conducted monthly to evaluate the public views of the ACA [10]. Briefly, the Kaiser poll is a random telephone dial sample (both landline and cell) of approximately 1000 to 1500 persons annually aged 18 years and older residing in the United States. The poll collects basic information on sociodemographic characteristics, health, and, relevant to our study, specifically asks respondents: “As you may know, a health reform bill was signed into law in 2010. Given what you know about the health reform law, do you have a generally favorable or unfavorable opinion of it?” The response set for this questions consists of: (1) favorable, (2) unfavorable, and (3) don’t know/refused.

From the Kaiser poll data, we determined the percent of respondents who reported being favorable versus unfavorable toward the ACA by month. Kaiser data were not available for 5 months (December 2012, January 2013, May 2013, July 2013, and August 2014).

### Analyses

Descriptively, we sought to examine the influence of key events regarding the ACA implementation on public response. Therefore, we identified the following historical events that took place during the study period (see Table 1 for details): three Supreme Court cases regarding the ACA; the reelection of President Obama (November 6, 2012), the first exchange open enrollment period (from October 1, 2013 to March 31, 2014), the start date for major expansions of health insurance coverage through the ACA (January 1, 2014), and the second exchange open enrollment period (from November 15, 2014 to February 15, 2015).

Across calendar months, we used Spearman correlation to evaluate for associations between public response measured using ACA tweets and the Kaiser poll. For instance, we compared the percentage of unfavorable ACA tweets per month with the percentage of Kaiser poll respondents who were unfavorable toward the ACA. As young adults tend to use Twitter more than older adults, we also examined correlations stratified by the age of Kaiser poll respondents [25]. Finally, we determined how the overall volume of ACA tweets throughout the study period varied over time.

To determine whether Americans were referring to the ACA as “Obamacare” more or less over time, we show the volume of ACA-related tweets that do and do not contain this term. For all analyses, we used R statistical software (R Foundation for Statistical Computing, Vienna, Austria). A 2-sided *P* value of less than .05 was considered statistically significant.

We also performed a subanalysis to test the robustness of the associations we observed to determine whether tweets from political and special interest groups impacted our results. To do so, we reanalyzed associations after excluding the 310,862 clearly political ACA tweets that included hashtags such as #gop (Grand Old Party), #teaparty, #p2 (Progressive 2.0), #PJNET (Patriot Journalist Network), #tlot (Top Libertarians on Twitter), #ccot (Christian Conservatives on Twitter), and #tcot (Top Conservatives on Twitter).

Finally, to provide some insight into the content of favorable versus unfavorable ACA tweets, we calculated the most frequently used other words (ie, not used to identify the tweets as ACA-related) and displayed these using word clouds.

## Results

### Comparison of Public Response Using Tweets to the Kaiser Poll

**Figure 1:**
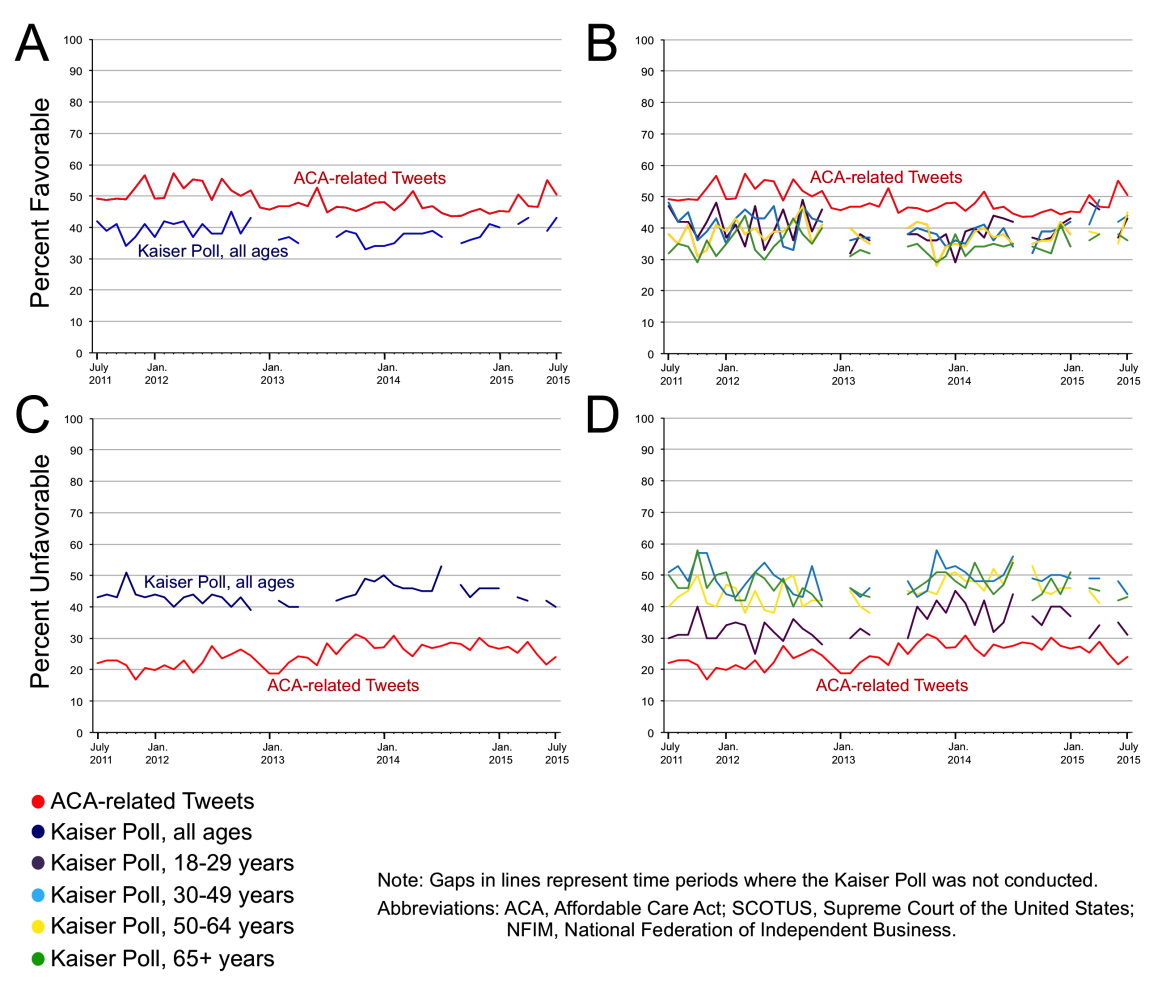
Favorable (A and B) versus unfavorable (C and D) public response to the Affordable Care Act using Tweets compared to results from the Kaiser Poll.

Figure 1 displays the percent favorable and unfavorable ACA-related tweets over time compared with public response measured by the Kaiser poll. Gaps in the Kaiser lines are time periods where the poll was not conducted. Approximately 50% of tweets were favorable compared with approximately 40% of Kaiser respondents being favorable toward the ACA throughout the time period (Figure 1). According to specific age categories, the percent of favorable Kaiser poll respondents differed little over time. Over the 5-year time period, approximately 20% of ACA tweets were unfavorable compared with 40% of Kaiser respondents being unfavorable toward the ACA . Across age categories, older Kaiser respondents were more likely to be unfavorable toward the ACA (eg, approximately 50% of adults aged 65 years and older reported being unfavorable toward the ACA).

In spite of these differences our Twitter-based measure of public opinion track results of the Kaiser poll quite well over time. The correlation coefficient between percentage of unfavorable ACA tweets and Kaiser respondents was .43, *P* value=.01 over the study time period. Likewise, the correlation coefficient between percentage of favorable ACA tweets and Kaiser respondents was .37, *P* value=.02 (Table 3).

Because Twitter users are likely to be younger than average, we also compared the percentage of favorable and unfavorable tweets according to Kaiser respondent age group. The strongest correlation was for unfavorable public response among Kaiser respondents between 18 and 29 years of age—a correlation coefficient of .47, *P* value=.01 (Table 3). The correlation coefficients for both favorable and unfavorable response between the two approaches were approximately .4, *P* value=.01 among Kaiser respondents between 30 and 49 years of age. Correlations were weak and statistically insignificant among older Kaiser respondents. These correlations persisted in our subanalysis where we remove tweets with political and special interest hashtags.

### The Volume of ACA Tweets Over Time

**Figure 2:**
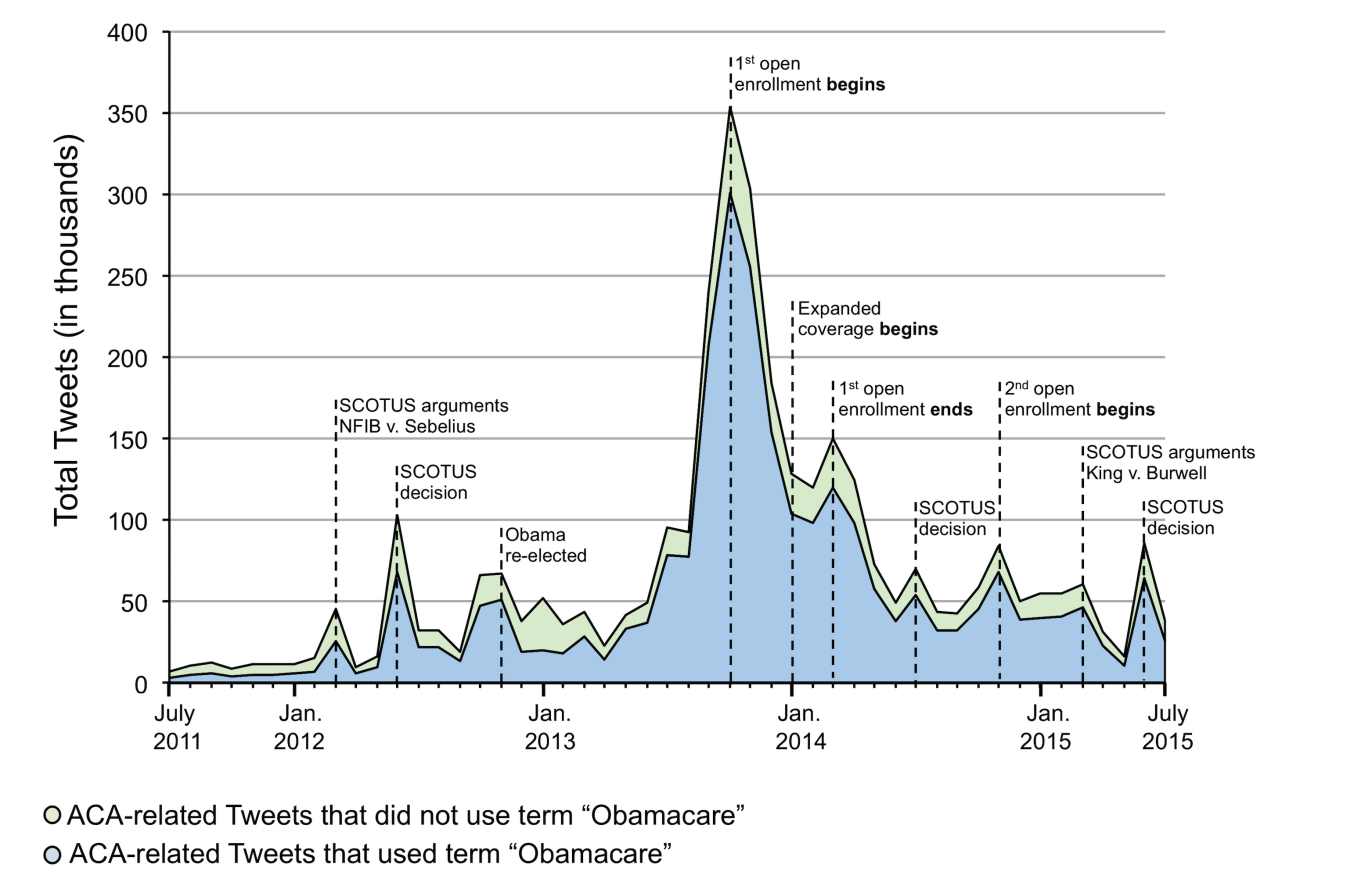
Total number of Affordable Care Act-related Tweets per month from July 2011 to January 2015.

Whereas public opinion may not change (much) in response to significant events in the ACA’s history, the volume of ACA-related tweets certainly does (Figure 2). Overall, the number of ACA-related tweets peaked during the first open enrollment period. In particular, the single month with the largest amount of ACA-related tweets was October 2013 (a total of 353,890 ACA tweets) —the beginning of the first open enrollment period. Other notable events such as the Supreme Court of the United States decision in June 2012 and beginning of the second open enrollment period in November 2014 also led to sharp spikes in ACA tweets. The term “Obamacare” was used in the great majority of ACA-related tweets throughout this period, with no evidence that this term became more or less common over time.

**Table 3:** Correlation between percentage of favorable (or unfavorable) tweets and percentage of favorable (or unfavorable) Kaiser poll respondents about the Affordable Care Act.

Kaiser respondents		Spearman correlation coefficient (*P* value)		
		Favorable	Unfavorable
All		.37 (.02)	.43 (.01)
By age category			
	18-29 years	.14 (.36)	.47 (.01)
	30-49 years	.41 (.01)	.40 (.01)
	50-64 years	.21 (.21)	.12 (.43)
	65+ years	.22 (.17)	.08 (.59)

### The Language of Favorable Versus Unfavorable ACA Tweets

**Figure 3:**
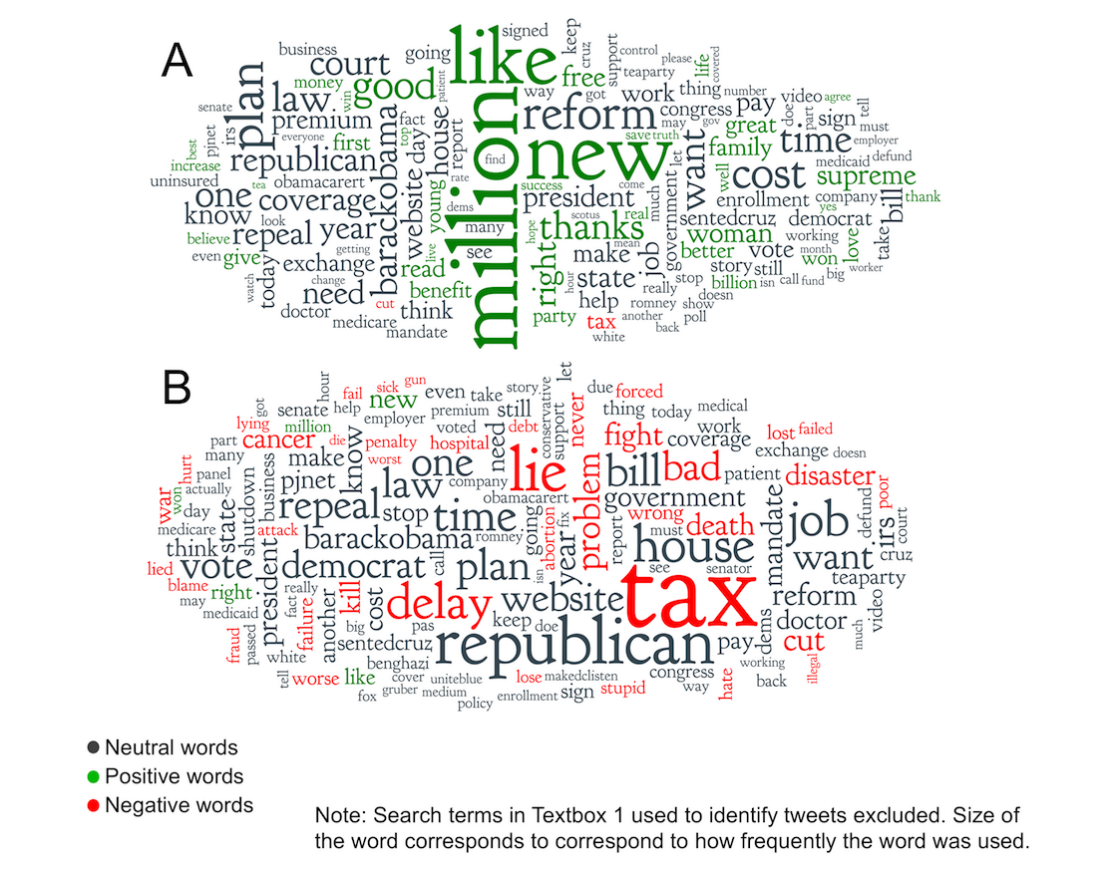
Common words used in favorable (A) versus unfavorable (B) Affordable Care Act-related Tweets.

Finally, in order to shed some light on the content of favorable and unfavorable ACA-related tweets, we tabulated the words most commonly used in each type of tweet, excluding the search terms in Textbox 1 that were used to identify ACA-related tweets initially. The results are presented as word clouds, an admittedly unscientific but nonetheless entertaining graphic tool in which size of the word corresponds to correspond to how frequently the word was used (Figure 3). A few of the most common words used in favorable ACA-related tweets included “like,” “million,” and “new.” Unfavorable ACA-related tweets included a more eclectic mix of words such as “tax,” “lie,” and “delay.”

## Discussion

### Principal Findings

To our knowledge this is the first study to compare Twitter-based measures of public opinion regarding the ACA to traditional polling results. Overall, we found evidence that Twitter data can be effectively leveraged to estimate public opinion, including the response (or lack of response) of opinion to specific events such as health care reform. Trends in the overall public response measured by sentiment of tweets paralleled the results of Kaiser poll, and the levels of favorable and unfavorable response were quite similar over time. Not surprisingly, public response to the ACA on Twitter correlated most highly with polling data for younger adults —the age group most likely to use social media platforms [25]. For policymakers interested in tracking public response, our results suggest that Twitter data can provide a less costly and more immediate alternative to traditional opinion polling, particularly for younger Americans. Examining the text of tweets themselves can offer insight into public opinion (or perhaps, the language used by those wishing to influence public opinion) beyond that of traditional polls. For instance, unfavorable tweets used language regarding taxation, dishonesty, and other negative terminology. Words in tweets also hinted at political affiliations associated with tweeting about the ACA, for example, “republican,” “democrat,” and “teaparty” were commonly used in ACA tweets.

The most striking finding may be that our Twitter-based measures of ACA public response exhibit the same remarkable stability over time that characterizes results from the Kaiser poll. One of the central puzzles about public opinion toward the ACA—why are opinions changing so little over time, even as major components of the law have been implemented and provided health insurance coverage to millions of Americans?—is just as pronounced in the immediate-response world of social media as in the more staid world of traditional opinion polling. Public opinion on Twitter toward the ACA could be more volatile, or more malleable, than opinions measured by the Kaiser poll—but they aren’t. The lack of significant change in favorable or unfavorable views toward the ACA over time does not mean people aren’t paying attention. On the contrary, they are not only paying attention, they are also expressing opinions in response to key events as we identified large changes in the volume of ACA tweets over time. The most striking spike in ACA tweets was in response to the first open enrollment period in October 2013 (during which the exchange enrollment website healthcare.gov failed to function properly). These large changes in volume, coupled with the lack of concomitant change in the favorable or unfavorable nature of the sentiments being addressed, echoes the thesis first advanced by Iyengar and Kinder that news may not so much change opinions as change how they are expressed [26]. In the age of social media, significant changes in the number of Americans expressing themselves in the absence of lack of change in public opinion might be reframed by saying that events in the real world do not change opinions, but they do change how often they are expressed.

There is growing use of Twitter to quantify public response. For instance, the sentiment expressed in tweets detected using either automated or manual annotation has been used to measure public response to vaccinations [27,28], the “Internet of things” [29], issues regarding electronic cigarettes [30], and climate change [31]. Collectively, this growing body of evidence points to encouraging findings regarding the use of Twitter as real-time barometer of collective public attitude. Our study contributes to several other studies that specifically used Twitter data to measure public response to health care reform [17-19,32]. Most similar to our study, King and colleagues used over 120,000 tweets related to the health and social care bill passed through parliament in England to examine public response [17]. They too found large spikes in the volume of tweets related to key events as the legislation was passed and moderate evidence of sentiment of tweets being correlated with public response. In a more recent report by Wong and colleagues, sentiment of ACA-related tweets were examined in relation to state-level health insurance marketplace enrollment [19]. After geocoding nearly 450,000 ACA-related tweets, the authors found a moderate association between ACA-related tweet sentiment and state-level enrollment. Our findings offer further support to the use of Twitter to quantify public response to health care reform as it correlates to some degree with a large national, ongoing poll. Furthermore, our findings of large spikes of tweets in relation to key events parallels findings observed in other studies (eg, [17,27,31,33]); in particular, we found significant fluxes in the amount of tweeting in the absence of swings in public opinion [17,26].

### Study Limitations

The chief limitation of Twitter data is that Twitter users are, by definition, not representative of the general population. Any ways in which Twitter users are different from the typical American—for example, being younger, more tech-savvy, or having a different political orientation—could bias our Twitter-based estimate of ACA sentiment, if these underlying differences also affect attitudes toward the ACA. This is the reason why our analysis begins by comparing our estimates with estimates from the nationally-representative Kaiser poll. Other limitations include the fact that our algorithms for identifying ACA-related tweets and for encoding the sentiments they contain could introduce systematic bias. Whereas these methods represent the current state of the art in social media analysis, this relatively young field is evolving rapidly and subsequent methodological refinements may improve on the approach we use here.

### Conclusions

In this study we found some evidence that Twitter may be useful for tracking public opinion regarding US health care reform as it appears to be comparable with conventional polling results. Similar to previous studies that used Twitter to measure public response, we found large changes in the amount of tweets in relation to key events; yet, during these time periods public opinion appeared to changed very little. Thus, the overall amount of tweets may also provide a potential indication of general public interest of a particular issue at any point in time. Whereas use of social media data for tracking public opinion is not without limitations, it is inexpensive, immediate, and can offer contextual insights beyond that of conventional polling.

## Abbreviations

ACA: Affordable Care Act
API: application programming interface
